# HLA Class II Antigens and Their Interactive Effect on Perinatal Mother-To-Child HIV-1 Transmission

**DOI:** 10.1371/journal.pone.0126068

**Published:** 2015-05-06

**Authors:** Ma Luo, Joanne Embree, Suzie Ramdahin, Thomas Bielawny, Tyler Laycock, Jeffrey Tuff, Darren Haber, Mariel Plummer, Francis A. Plummer

**Affiliations:** 1 Department of Medical Microbiology, University of Manitoba, Winnipeg, Manitoba, Canada; 2 National Microbiology Laboratory, Winnipeg, Manitoba, Canada; Centro di Riferimento Oncologico, IRCCS National Cancer Institute, ITALY

## Abstract

HLA class II antigens are central in initiating antigen-specific CD4+ T cell responses to HIV-1. Specific alleles have been associated with differential responses to HIV-1 infection and disease among adults. This study aims to determine the influence of HLA class II genes and their interactive effect on mother-child perinatal transmission in a drug naïve, Mother-Child HIV transmission cohort established in Kenya, Africa in 1986. Our study showed that DRB concordance between mother and child increased risk of perinatal HIV transmission by three fold (*P* = 0.00035/*P*c = 0.0014, OR: 3.09, 95%CI, 1.64-5.83). Whereas, DPA1, DPB1 and DQB1 concordance between mother and child had no significant influence on perinatal HIV transmission. In addition, stratified analysis showed that DRB1*15:03+ phenotype (mother or child) significantly increases the risk of perinatal HIV-1 transmission. Without DRB1*15:03, DRB1 discordance between mother and child provided 5 fold protection (*P* = 0.00008, OR: 0.186, 95%CI: 0.081-0.427). However, the protective effect of DRB discordance was diminished if either the mother or the child was DRB1*15:03+ phenotype (*P* = 0.49-0.98, OR: 0.7-0.99, 95%CI: 0.246-2.956). DRB3+ children were less likely to be infected perinatally (*P* = 0.0006, *Pc* = 0.014; OR:0.343, 95%CI:0.183-0.642). However, there is a 4 fold increase in risk of being infected at birth if DRB3+ children were born to DRB1*15:03+ mother compared to those with DRB1*15:03- mother. Our study showed that DRB concordance/discordance, DRB1*15:03, children’s DRB3 phenotype and their interactions play an important role in perinatal HIV transmission. Identification of genetic factors associated with protection or increased risk in perinatal transmission will help develop alternative prevention and treatment methods in the event of increases in drug resistance of ARV.

## Introduction

Since the beginning of the HIV/AIDS epidemic, HIV-1 has infected more than 60 million people and caused more than 25 million deaths in the world [1]. Because of transmission from infected mothers to their infants, HIV-1 infection is also one of the major causes of childhood death globally. An estimated 3.34 million children under 15 years old are living with HIV/AIDS [1]. Each day, over 700 children become infected with HIV in 2012, the vast majority of whom are newborns [1]. The majority of pediatric HIV-1 infections occur through mother-to-child transmissions (MCHT) perinatally and through breast feeding [2]. Amazingly even though the chances of transmitting HIV-1 infected cells and free viruses from an infected mother to a child is very high, not all children born to HIV-infected mother are infected with the virus. Without anti-retroviral (ARV) drug treatment, from 16% to 30% of the children born to HIV-infected mother are infected perinatally thus between 70% to 84% of the children escape HIV-1 infection [1]. Better understanding of the factors influencing MCHT will improve our knowledge of the mechanism of transmission, and thus enable the development of alternative interventions in addition to ARV for transmission prevention.

High plasma HIV-1 load, low maternal CD4 cell counts, vaginal delivery, low gestational age, non-use of ARV by mother and/or neonates, and breast feeding have all been identified as independent factors associated with MCHT [2–4]. Host genetic factors also play an important role [5–9]. HLA class I concordance between mother and child increases the risk of HIV transmission [6,9,10] perinatally but not through breast feedings. This is thought to be largely due to alloimmunity generated by the host-antigen associated pathogens that carry host antigens on their surface during transmission between individuals [11]. The host proteins including HLA molecules on the surface of HIV-1 infected cells and envelope of HIV virus masked the virus and allowed the HIV-1 virus or infected cells to avoid detection by the host immune system. While this enables HIV-1 to maintain a chronic infection in the infected host, the host antigens expressed on their surface work against them during transmission between different hosts. This is because, among host antigens, the polymorphic HLA induces the most rapid and potent prophylactic immune reaction known against intraspecific antigens. Thus, the HLA molecules on the surface of HIV-1 infected cells and HIV virus can prevent transmission through induction of prophylactic alloimmune responses due to the mismatch of HLA between individuals of different HLA alleles. Alloimmunity due to discordance of HLA class I alleles between mother and child likely plays an important role in preventing perinatal HIV-1 transmission in the absence of antiretroviral treatment [6,9,10].

Specific HLA class I genotypes appear to influence MCHT [12]. HLA class II antigens are also very diverse and consist of DRB (1, 3, 4, and 5), DQA1, DQB1, DPA1, and DPB1. More than 2870 class II alleles have been identified and DRB1 is the most polymorphic class II gene with more than 1642 identified alleles [13,15]. HLA class II antigens play an important role in response to infectious and autoimmune diseases through presenting pathogenic and self-peptides to CD4+ T cells. Matching HLA class II antigens between unrelated donor and recipient in transplantation improves survival [16–19] indicating that alloimmunity by class II antigens should not be ignored. HLA class II antigens present pathogenic peptides to CD4 T cells and play an important role in resistance/susceptibility to HIV-1 infection [20–22]. However, the effect of HLA class II antigens on mother-child HIV-1 transmission has not been well studied. A lone study on the effect of class II antigens yielded inconclusive results due to a small number of HIV-1 positive children in the study and the confounding effect of antiretroviral treatment [6]. A thorough investigation of the influence of HLA class II antigens on MCHT is important to fully understand the risk/protective factors in perinatal HIV transmission and to develop alternative methods to ARV for the prevention of transmission in the event of development of drug resistance, which will make ARV interventions ineffective.

The Mother-child HIV Transmission cohort in Nairobi was established in 1986 at the Pumwani Maternal Hospital to study risk factors in mother-child HIV-1 transmission [23]. In this cohort overall,

16% of children were infected perinatally. Previous studies in this cohort showed that HLA class I concordance between mother and child increases the risk of HIV transmission and that specific HLA class I supertypes influence mother-child HIV-1 transmission [5,10]. The protective effect of class I discordance between mother and children was also seen in two other cohort studies [6,9]. This study was designed to investigate the influence of HLA class II antigens and their interactive effect on mother-child HIV transmission, we studied 112 perinatally infected children and 187 uninfected controls from the cohort and genotyped HLA-DRB (includes DRB1, DRB3, BRB4 and DRB5), DQB1, DPA1 and DPB1 genes of the mother and the children using a high-resolution sequence-based method [24,25]. Since the population was antiretroviral drug naïve at the time of the infant birth, the effect of genetic factors, such as HLA class II are not confounded by ARV therapy which significantly reduces perinatal transmission [3].

## Materials and Methods

### Study population

A subset of HIV-1 infected mothers and their children enrolled in the University of Nairobi HIV-1 Perinatal Transmission and Pediatric AIDS Study from 1986 to 2000 were included in this study. Its enrolment protocol and study design have been previously described [23]. All mothers and children examined in this study were antiretroviral treatment naïve. Every child was vaginally delivered and most at term. Around 16% of children were determined to be infected perinatally in the cohort. Breast-feeding was advocated according to the World Health Organization breast-feeding guidelines at the time of enrolment. Blood for HIV-1 serologic assays was obtained at birth, at 6, 14, and 24 weeks, and every 3 months thereafter. Blood for HIV-1 provirus PCR was collected. Children who born to infected mothers, become seronegative and remained PCR negative were considered uninfected. Even though in this cohort overall only

16% of children were infected perinatally, to obtain nearly equal number of perinatally infected and uninfected children for this study to increase the statistical power we tried to include as many mothers with perinatally infected children as we could find in this cohort and randomly matched them with close to equal numbers of mothers with uninfected children.

Among the 299 children born to HIV positive mothers included in this study, 187 were HIV uninfected and 112 became HIV infected perinatally. The classification of children’s HIV status and HIV serology and PCR testing were reported in previous publications [5,10]. This study has been approved by the Ethics Committee of the University of Manitoba and the Ethics and Research Committee of Kenyatta National Hospital. Informed consent was obtained from all women enrolled in the study.

### Typing of HLA class II genes

A high-resolution sequence-based typing method was used to type HLA class II genes [24,25]. Genomic DNA was isolated from frozen blood, PBLs, buffy coats, or blood spots of HIV positive mothers and their children. HLA DRB1, 3, 4 and 5, DPA1, DPB1, and DQB1 genes were amplified with gene specific primers (Table 1). Agarose gel electrophoresis was used to confirm the amplification of the class II genes. The amplified PCR products were purified with Millipore PCR purification system and sequenced with specific primers using BigDye 1.0 cycle sequencing kits. The sequencing products were analyzed with ABI3100 Genetic Analyzer. The class II sequences were typed using CodonExpress, a computer software program developed based on a Taxonomy-based Sequence Analysis [24,25]. DPA1, DPB1, and DQB1 were typed at the 4 digit resolution. DRB1 was typed to the group level (2-digit resolution) for alleles in DRB1*03, DRB1*04, DRB1*08, DRB1*11, DRB1*12, DRB1*13, and DRB1*14. Four digit allele level resolution was achieved for DRB1*01:01, DRB1*01:02, DRB1*07:01, DRB1*10:01, and DRB1*15:03. The DRB3, DRB4 and DRB5 were typed at two digit level. All alleles are reported at 4 digit resolution except for the ones were typed only at the group level.

**Table 1:** PCR and sequencing primers used for genotyping HLA class II genes.

name	specificity	primer sequence
**DQB1**		
DQBDNAF	5'DQB1 (PCR forward primer)	5'-TCCCCGCAGAGGATTTCGTG-3'
DQBDNAR	3'DQB1 (PCR reverse primer)	5'-GGCGACGACGCTCACCTC-3'
DQBSEQ1	DQB1 Sequencing primer (sense)	5'-GCAGAGGATTTCGTGTTCCAG-3'
DQBSEQ3	DQB1 Sequencing primer (antisense)	5'-CCTTCTGGCTGTTCCAGTACTC-3'
**DRB**		
DRBPRCF	5'-DRB	5'-GTTCGTGTCCCCACAGCACGTTTC-3'
DRBPCRR	3'-DRB	5'-CATGCTCACCTCGCCGCTGCAC-3'
DRBSEQ4	DRB (antisense)	5'-GAAGCTCTCACCAACCCCGTAG-3'
DRB01SEQ	DRB1*01s	5'-TTGTGGCAGCTTAAGTTTGAA-3'
DRB031234SEQ	DRB1*03s, 11s, 12s, 13s, 14s	5'-CACGTTTCTTGGAGTACTCTAC-3'
DRB04SEQ	DRB1*04s	5'-CCTGGACAGATACTTCTATC-3'
DRB08SEQ	DRB1*08s	5'-TTCTTGGAGTACTCTACGG-3'
DRB1516SEQ	DRB1*15s, 16s	5'-CACGTTTCCTGTGGCAGCCTAAGA-3'
DRB3SEQ	DRB3	5'-CCACAGCACGTTTCTTGGAGCT-3
DRB4SEQ	DRB4	5'-GAGCGAGTGTGGAACCTGATC-3'
DRB5SEQ	DRB5 and DRB1*09	5'-CACGTTTCTTGCAGCAGGA-3'
DRB86SEQ	DRB with GTG at codon 86	5'-CTGCACTGTGAAGCTCTCAC-3'
DRB58SEQ	DRB with GAG at codon 58	5'-AGCTGGGGCGGCCTGATGAG-3'
DRB26SEQ	DRB with TAC at codon 26	5'-TGGGACGGAGCGGGTGCGGTA-3'
**DPA1**		
DPAPCRF	5'DPA1	5’- ACATTTTGTCGTGTTTTTCTCT -3’
DPAPCRR	3'DPA1	5’- GTTGACCTTCCCTACTCTC -3’
DPASEQF	DPA1 Sequencing primer (sense)	5’- GGCGGACCATGTGTCAACTTAT -3
DPASEQR	DPA1 Sequencing primer (antisense)	5’- GCAAGGTTGGTGTGAGTCCG-3’
**DPB1**		
DPBPCRF	5'DPB1	5’-GAGAGTGGCGCCTCCGCTCAT-3’
DPBPCRR	3'DPB1	5’-GCCGGCCAAAGCCCTCACTC-3’
DPBSEQF	DPB1 Sequencing primer (sense)	5’- CCTCCCCGCAGAGAATTAC-3’
DPBSEQR	DPB1 Sequencing primer (antisense)	5’-GAGGTGAGTGAGGGCTTTG-3’

### Classification of mother-child HLA matching

The number of matching between mother’s and child’s HLA class II type is assessed following the method reported in the previous publication [10]. Specifically, because for each class II gene, a child inherits one allele from mother and one from father, and will match at least 50% of mother’s class II alleles. If the two alleles of the child at a class II locus match the two alleles of the mother, they are considered concordant at that locus. If the mother is homozygous at a locus, she is also considered to be concordant at that locus with her child because to the child there is no foreign antigen.

### Statistical analysis

The effect of HLA class II genes on mother-child HIV transmission was assessed by statistical analysis with SPSS 13.0. The standard univariate Fisher’s exact test (*P* value, odds ratio, confidence interval 95%) were utilized to determine the relationship between binary outcomes and explanatory variables. A total of 26 class II alleles or allele groups with a population frequency of 4% or higher were analyzed for the associations with risk of transmission. Logistic regression was used for multivariate analysis. False-discovery rate (FDR) was used to adjust P value for multiple comparisons and the p values of 26 class II allele or allele groups with frequencies above 4% were used for FDR calculation. FDR was calculated using the FDR calculator in Excel file hosted at Rowett Research Institute website (*http*:*//www*.*rowett*.*ac*.*uk/~gwh/fdr*.*html*).

## Results

### Biology of HIV positive mothers and their children

A total of 299 children born to 234 mothers were examined in this study. It included 51 mothers with two or more children. Since many factors might influence MCHT, we compared the biology of mothers who have only uninfected children with mothers who have one or more HIV-1 positive children for factors such as age, marital status, duration of gestation and labor, CD8+ and CD4+ counts, as well as the percent of CD4+ and CD8+ T cells at the time of delivery (Table 2). We observed no significant difference in the factors examined between these two groups of women within the study period. We do not have viral load information for these women due to sample storage conditions in Kenya and the absence of reliable viral load testing methods during the period of study. However, studies showed that CD4+ counts and the percentage of CD4+ T cells of the patients infected with HIV-1 are negatively correlated to the viral load [26,27] and analysis of the correlation of log viral load with CD4+ T cell counts and the percent of CD4+ T cells in a group of drug naïve Kenyan women also showed a linear correlation (Multiple R:0.921, R^2^: 0.848), the similar CD4+ counts for both groups of women in the absence of anti-retroviral treatment (>500) suggest that they are healthy and with comparable viral load at the time of this study. We also compared biological markers of HIV-1 infected children with those who were HIV-1 negative, such as sex, birth weight, and gestation age, as well as duration of follow-up (Table 2). The sex ratio, birth weight, and gestational age are very similar between the two groups of children, except that the follow-up time of HIV-1 negative children is almost double that of the HIV-1 positive children. In summary, during the study period there is no significant difference in the examined biological markers of the mother and children in this study.

**Table 2:** Comparison of demographic, obstetric, and other characteristics of mothers and children in the study.

**Biology factors**	**mother**	**N**	**Mean**	**Std. Deviation**	**Std. Error Mean**	**p value**
						**(2-tailed)**
age	only have HIV- Kids	135	23.21	4.22	0.36	0.579
	have HIV+ Kids	97	22.9	4.17	0.42	
MARIT_STAT	only have HIV- Kids	134	2.54	0.83	0.07	0.755
	have HIV+ kids	98	2.57	0.81	0.08	
gravida	only have HIV- Kids	134	2.64	1.63	0.14	0.301
	have HIV+ kids	97	2.41	1.7	0.17	
GESTAGE_WK	only have HIV- Kids	126	38.52	4.13	0.37	0.97
	have HIV+ kids	94	38.54	2.86	0.3	
GESTAGE_AGE	only have HIV- Kids	103 (90.5%)				NS
**≥** 37 weeks	have HIV+ kids	75 (91.5%)				
SEXPART_5	only have HIV- Kids	132	3.1	9.14	0.8	0.951
	have HIV+ kids	96	3.18	10.03	1.02	
RUPTURED_H	only have HIV- Kids	130	4.05	5.61	0.49	0.546
	have HIV+ kids	90	3.58	5.95	0.63	
LABOR_HRS	only have HIV- Kids	132	11.01	8.83	0.77	0.702
	have HIV+ kids	90	10.61	5.15	0.54	
CD4+ count	only have HIV-Kids	116	530.8	261.7	24	0.598
	have HIV+ kids	90	563.0	530.2	56	
CD4+ (%)	only have HIV- Kids	116	24.57	0.08	0.007	0.058
	have HIV+ kids	90	22.24	0.096	0.01	
CD8+ count	only have HIV- Kids	116	1102	529.2	50	0.356
	have HIV+ kids	89	1225	978.3	87	
CD8+ (%)	only have HIV- Kids	116	50.22	10.03	0.92	0.989
	have HIV+ kids	89	50.2	12.56	1.32	
**Biology factors**	**children**	**N**	**Mean**	**Std. Deviation**	**Std. Error Mean**	**p value**
						**(2-tailed)**
Birth weight (gram)	HIV-	152	3028	545.2	44.2	0.686
	HIV+	97	3057	587.2	59.6	
Sex	HIV-	173	1.54	0.5	0.04	0.436
	HIV+	107	1.5	0.5	0.05	
Duration of follow-up (month)	HIV-	169	46.52	45.33	3.48	0.00002
	HIV+	105	24.87	30.72	3.00	

### The major HLA class II alleles and allele groups of the study population

High resolution sequence-based typing identified 13 DPA1, 42 DPB1, 18 DQB1 alleles in 234 mothers. Higher number of alleles were identified in the 298 children (13 DPA1, 47 DPB1 and 19 DQB1 alleles). Ten DRB1 allele groups were identified by a low resolution sequence-based DRB typing in both mothers and their children. The alleles or allele groups with frequencies above 4% of the population are listed in Table 3. In general the frequencies of the major HLA class I allele/allele groups of mothers and their children are similar, except that the DPB1*17:01 phenotype frequency of children is significantly higher than that of the mothers (12.08% vs 6.41%, *p* = 0.019), and the mothers’ DRB1*08 phenotype frequency is significantly higher than that of the children (15.81% vs 9.73%, *p* = 0.048).

**Table 3:** Major HLA class II alleles/allele groups in mothers and their children.

	mother	children	p value
	n = 234	n = 298	uncorrected
**DPA1**			
01:03	125(53.42%)	159(53.36%)	ns
02:01	63(26.92%)	101(33.89%)	ns
02:02	74(31.62%)	91(30.54%)	ns
03:01	79(33.76%)	106(35.57%)	ns
04:01	11(4.70%)	12(4.03%)	ns
**DPB1**			
01:01	95(40.60%)	115(38.59%)	ns
02:01	60(25.64%)	66(22.15%)	ns
03:01	27(11.54%)	37(12.42%)	ns
04:01	18(7.69%)	23(7.72%)	ns
04:02	80(34.19%)	90(30.20%)	ns
13:01	15(6.41%)	20(6.71%)	ns
**17:01**	**15(6.41%)**	**36(12.08%)**	**0.019**
18:01	19(8.12%)	15(5.03%)	ns
55:01	10(4.27%)		
**DQB1**			
02:01	62(26.50%)	79(26.51%)	ns
03:01	85(36.32%)	109(36.58%)	ns
04:02	39(16.67%)	46(15.44%)	ns
05:01	60(25.64%)	78(26.17%)	ns
06:02	62(26.50%)	88(29.53%)	ns
06:04	18(7.69%)	19(6.38%)	ns
06:09	18(7.69%)	17(5.70%)	ns
**DRB1**			
01	24(10.26%)	36(12.08%)	ns
03/13/14	150(64.10%)	184(61.74%)	ns
04		15(5.03%)	ns
07:01	21(8.97%)	33(11.07%)	ns
**08**	**37(15.81%)**	**29(9.73%)**	**0.048**
10:01	14(5.98%)	19(6.38%)	ns
11	98(41.88%)	126(42.28%)	ns
15:03	55(23.50%)	62(20.81%)	ns
DRB3	188(80.34%)	250(83.89%)	ns
DRB4	35(14.96%)	47(15.77%)	ns
DRB5	55(23.50%)	61(20.47%)	ns

### DRB concordance increased the risk of perinatal HIV–1 transmission

Because polymorphic HLA induces the most rapid and potent prophylactic immune reaction known against intraspecific antigens and previous studies have shown that concordance of HLA class I genes increases risk of mother-child HIV transmission, we analyzed the effect of concordance of HLA class II genes on mother-child perinatal HIV transmission. The analysis showed that concordance of DRB genes between mother and child increased the risk of perinatal transmission of HIV-1 by 3 fold (Table 4). A significantly higher percentage of HIV-1 perinatally infected children are DRB concordant with their mothers, while significantly higher percentage of perinatally uninfected children are DRB discordant (p = 0.00035, odds ratio: 3.09, 95%CI:1.64–5.83). Whereas, concordance or discordance of DPA1, DPB1 and DQB1 between mother and child had no significant influence on perinatal HIV-1 transmission (*p* = 0.618, *p* = 0.213, and *p* = 0.758 respectively). We stratified Mother’s CD4+ and CD8+ T cell counts based on the concordance of class II genes between mother and child, and observed no significant difference (data not shown). The increased perinatal HIV transmission appears to be correlated with DRB concordance between mother and child, and not the mother’s CD4+ T cell counts.

**Table 4:** The effect of HLA class II concordance on perinatal HIV-1 transmission.

	HIV+ children (n = 92)	HIV- children (n = 162)	p value(corrected)	odds ratio (95%CI)
children DRB concordant with mother	29 (31.95%)	21 (12.96%)	0.00035(0.0014)	3.09 (1.64–5.83)
	(n = 96)	(n = (169)		
children DPA concordant with mother	43 (44.8%)	67 (39.6%)	n.s	1.24 (0.74–2.05)
	(n = 95)	(n = 159)		
children DPB concordant with mother	24 (25.3%)	34 (21.4%)	n.s	1.24 (0.68–2.26)
	(n = 80)	(n = 160)		
children DQB concordant with mother	27(33.8%)	57(35.6%)	n.s	0.92 (0.52–1.62)

### The effect of Mother’s and children’s HLA class II genotype and perinatal HIV-1 transmission

We examined the effect of several previously reported HLA class II alleles associated with resistance or susceptibility to HIV-1 infection in the Pumwani Sexworker cohort on perinatal HIV-1 transmission [20–22]. The influence of specific class II genotypes on perinatal HIV-1 transmission was also examined for those with frequencies 4% or higher in the study population. Several class II genotypes of the mothers or children were significantly associated with either protection or increased risk of perinatal transmission (Table 5). Mothers with DPB1*55:01 were 5 times more likely to perinatally transmit HIV-1 virus to their children than mothers without DPB1*55:01 (*p* = 0.023, OR:5.1, 95%CI:1.06–24.57), while mothers with DQB1*03:03 only have HIV negative children (Table 5). Children with DRB3 phenotype were significantly less likely to be perinatally infected (p = 0.0006, OR: 0.304, 95%CI: 0.128–0.723), whereas children with DPA1*04:01 were more likely to be infected at birth (p = 0.036, OR:3.696, 95%CI:1.08–12.6). Multivariate analysis showed that both DRB discordance and children’s DRB3 phenotype are important at protecting children from perinatal HIV-1 infection (Table 6). Similarly, DRB concordance, mother’s DPB1*55:01 phenotype and children’s DPA1*04:01 phenotype are independent risk factors for HIV-1 transmission at birth (Table 6). Stratified analysis of CD4+ T cell counts based on mother’s HLA phenotypes observed no significant difference (data not shown). After correction for multiple comparisons only the protective correlation of children’s DRB3 phenotype remains significant (*Pc* = 0.014).

**Table 5:** The influence of specific HLA class II phenotypes on perinatal HIV-1 transmission.

**Mother**				
mother's class II Phenotype	mothers have only HIV negative kid (n = 127)	mothers have HIV positive kid (n = 106)	p value (FDR)	odds ratio (95%CI)
DPB1*55:01	2 (1.6%)	8 (7.5%)	0.027 (NS)	5.1 (1.06–24.57)
DQB1*03:03	6 (4.7%)	0	0.025 (NS)	do not transmit
**Children**				
Children's class II Phenotype	HIV-	HIV+	p value (FDR)	odds ratio (95%CI)
	(n = 187)	(n = 112)		
DPA1*04:01	4 (2.1%)	8 (7.1%)	0.036 (NS)	3.519 (1.04–11.97)
DRB3	167 (89.3%)	83 (74.1%)	0.0006 (0.014)	0.343 (0.183–0.642)

**Table 6:** Multi-variate analysis.

Binary logistic regression analysis							
Step 1	B	S.E.	Wald	Sig	Exp(B)	95%CI for Exp(B)	
						Lower	Upper
DRB_discordant	0.837	0.344	5.938	0.015	2.310	1.178	4.528
Children_DRB3	1.340	0.523	6.571	0.010	3.819	1.371	10.641
constant	-0.876	0.157	30.948	0.00000003	0.416		
DRB_concordant	-0.992	0.355	7.832	0.005	0.371	0.185	0.743
Mom_DPB1*5501	-1.978	0.826	5.726	0.017	0.138	0.027	0.699
Children_DPA1*0401	-1.272	0.648	3.850	0.05	0.280	0.079	0.999
constant	3.327	1.072	9.638	0.002	27.847		

### The interactive effect of DRB1*15:03, DRB concordance/discordance and DRB3 in perinatal HIV transmission

DRB1*15:03 has been identified as a risk factor to HIV-1 infection in the Pumwani Sexworker Cohort [21]. However, when examining the effect of the mother’s DRB1*15:03 genotype on perinatal HIV-1 transmission we did not see the association of DRB1*15:03 in mother or children with increased risk of perinatal HIV transmission (*p* = 0.334 and *p* = 0.232, respectively). Because of the strong effect of DRB discordance on reducing the risk of perinatal HIV transmission, it is possible that the effect of DRB1*15:03 is masked by the DRB discordance. To confirm this we compared HIV-1 infection in children who were born to DRB1*15:03+ mothers with those whose mother were DRB1*15:03- and examined DRB concordance/discordance on perinatal HIV transmission (Table 7). Among the children who were DRB discordant with their mothers being born to DRB1*15:03- mother their odds of being infected at birth is less than 20%. Whereas the children with DRB1*15:03+ mother the protective effect of DRB discordance disappeared and their odds of being infected at birth is close to 1 (Table 7). DRB1*15:03+ phenotype, mother or child, abolished the protective effect of DRB discordance (Table 7). We also examined whether the interactive effect can also be observed between DRB1*15:03 and DRB3. The results showed that children with DRB3 phenotype were significantly less likely to be infected at birth (Table 5), however, if DRB3+ children born to DRB1*15:03+ mother the odds of being infected at birth increased 4 fold compared to those with DRB1*15:03- mother (Table 7). Therefore, both DRB concordance and DRB1*15:03+ phenotype are risk factors in mother-child HIV-1 transmission.

**Table 7:** Interactive effect of DRB discordance, DRB1*1503 phenotype and specific class II alleles on perinatal mother-child HIV transmission.

	Children with DRB1*1503- mother (n = 191)				Children with DRB1*1503+ mother (n = 62)			
	HIV+ (n = 66)	HIV- (n = 125)	p value	odds ratio (95%CI)	HIV+ (n = 26)	HIV- (n = 36)	p value	odds ratio (95%CI)
DRB_ discord	45 (68.2%)	115 (92.0%)	0.00002	0.186 (0.081–0.427)	18 (69.2%)	25 (69.4%)	0.986	0.99 (0.332–2.956)
	DRB1*1503- children (n = 195)				DRB1*1503+ children (n = 56)			
	HIV+ (n = 66)	HIV- (n = 129)	p value	odds ratio (95%CI)	HIV+ (n = 24)	HIV- (n = 32)	p value	odds ratio (95%CI)
DRB_ discord	49 (74.2%)	119 (92.2%)	0.0006	0.242 (0.104–0.566)	14 (58.3%)	21 (65.6%)	0.577	0.733 (0.246–2.184)
	DRB1*1503- mother and children (n = 174)				DRB1*1503+ mother and children (n = 80)			
	HIV+ (n = 59)	HIV- (n = 115)	p value	odds ratio (95%CI)	HIV+ (n = 33)	HIV- (n = 47)	p value	odds ratio (95%CI)
DRB_discord	40 (67.8%)	105 (91.3%)	0.00008	0.201 (0.086–0.468)	10 (30.3%)	11 (23.4%)	0.490	0.703 (0.258–1.917)
	DRB1*1503- mother (n = 203)				DRB1*1503+ mother (n = 61)			
DRB3+ children	HIV+ (n = 71)	HIV- (n = 132)	p value	odds ratio (95%CI)	HIV+ (n = 25)	HIV- (n = 36)	p value	odds ratio (95%CI)
	66 (93.0%)	131 (99.2%)	0.012	0.101 (0.012–0.880)	16 (64.0%)	29 (80.6%)	0.148	0.429 (0.134–1.370)
	DRB1*1503- children (n = 214)				DRB1*1503+ children (n = 57)			
DRB3+ children	HIV+ (n = 75)	HIV- (n = 139)	p value	odds ratio (95%CI)	HIV+ (n = 23)	HIV- (n = 34)	p value	odds ratio (95%CI)
	69 (92.0%)	138 (99.3%)	0.004	0.083 (0.010–0.706)	14 (60.9%)	26 (76.5%)	0.207	0.479 (0.151–1.516)
	DRB1*1503- mother and children (n = 189)				DRB1*1503+ mother and children (n = 85)			
	HIV+ (n = 65)	HIV- (n = 124)	p value	odds ratio (95%CI)	HIV+ (n = 33)	HIV- (n = 49)	p value	odds ratio (95%CI)
DRB3+ children	61 (93.8%)	123 (99.2%)	0.048	0.124 (0.014–1.133)	22 (66.7%)	41 (83.7%)	0.109	0.390 (0.137–1.113)

## Discussions

In the absence of interventions, the risk of mother-to-child perinatal transmission of HIV is between 15–30% [1]. Among many factors influencing mother-child perinatal transmission, alloimmunity appears to play an important role to prevent a full bloom transmission of HIV-1 from mother to child [6,10]. This can be seen in the protective effect of DRB discordance between the mother and child in reducing the vertical HIV-1 transmission in our study. Among class II antigens, DRB1 is the most polymorphic locus with 1638 alleles identified to date [13–15, 28, 29]. In addition, DRB1 forms haplotypes with 3 other functional polymorphic DRB genes including DRB3, DRB4 and DRB5. This makes DRB gene cluster the most polymorphic complex. HLA class II antigens are constitutively expressed on the professional antigen-presenting cells and the level of expression of DRB is higher than DQ and DP [30,31]. Thus, the effect of alloimmunity generated by DRB mismatch between mother and child would be greater because its much higher polymorphism and the higher-level expression of DRB genes than that of other class II genes.

DRB1*15:03 was significantly associated with increased susceptibility to HIV-1 infection in the Pumwani Sex Worker Cohort [21]. In addition, DRB1*15:03 increased seroconversion in Zambian discordant couples [32] and DR2 (DRB1*15/16-DRB5 haplotype) was associated with susceptibility to HIV-1 infection in a South Indian cohort [33]. Our study showed that DRB1*15:03 genotype of mother or children is also a risk factor for perinatal HIV transmission. The DRB1*15:03+ phenotype of the mother or children abrogates the protective effect of alloimmunity due to DRB discordance. DRB1*15 is associated with susceptibility to a variety of autoimmune diseases such as aplastic anemia [33], multiple sclerosis [34], and rheumatoid arthritis [35]. It suggests that individuals with this HLA type tend to have a higher level of immune activation that could lead to higher rates of HIV-1 transmission due to an increased number of activated CD4+ T-cells, which are easy targets of HIV-1.

In addition to the protective effect of alloimmunity due to discordance of HLA class II DRB antigen in mother-child HIV-1 transmission and the increased risk of DRB1*15:03 phenotype in HIV transmission at birth, we also identified several other protective/susceptible class II phenotypes. Mothers with the DQB1*03:03 phenotype do not transmit HIV-1 to their children perinatally and the children with DRB3 genotype were significantly less likely to be infected during birth (Table 4). Whereas, mothers with DPB1*55:01 phenotype are more likely to have children infected perinatally and children with DPA1*04:01 phenotype have a higher risk to be infected during birth (Table 4). The associations of DPB1*55:01 (mother) and DPA1*04:01 (children) with increased risk of perinatal HIV-1 transmission are independent of DRB concordance as see in the multivariate analysis. After correction for multiple comparisons only the association of DRB3 genotype of the children remains significant. Therefore, the association of DPB1*55:01, DQB1*03:03 and DPA1*04:01 with differential risk of perinatal HIV transmission need to be confirmed in a different population. Alloimmunity is a possible explanation for the protective effect of children’s DRB3 genotype, since DRB3 forms haplotypes with a great diversity of DRB1 alleles, including DRB1*03, 11, 12, 13 and 14. The protective effect of DRB3 discordance was also observed in the HIV sero-discordant couples [36].

HLA class II antigens play an important role in response to infectious and autoimmune diseases through presenting pathogenic and self-peptides to CD4+ T cells. It is reasonable to assume that the 16 alleles of the 8 functional HLA class II genes in a given individual all play a role. The interactive effect of CD4+ T cell responses restricted by the 16 HLA class II alleles and the genetic diversity of these different HLA class II alleles could influence perinatal HIV transmission. In a previous study we have observed interactive effect of HLA class I alleles in HIV seroconversion and disease progression in the Pumwani sex worker cohort [37]. Our current study has shown the interactive effect of HLA class II genes on perinatal HIV transmission. The beneficial effect of alloimmunity by DRB discordance and DRB3 can be diminished by the detrimental effect of DRB1*15:03.

High plasma HIV-1 load, low maternal CD4 cell counts, vaginal delivery, low gestational age, non-use of ARV by mother and/or neonates, and breast feeding have all been identified as independent factors associated with MCHT [2–4]. We have compared CD4+ and CD8+ T cell counts, gestational age and all related biological factors between mothers who did not transmit the virus to their children perinatally with the mothers who have transmitted the virus during birth. We observed no significant difference in the factors examined between these two groups of women within the study period. Due to sample storage conditions in Kenya and the absence of reliable viral load testing methods during the period of study, we do not have viral load information for these women. However, studies showed that CD4+ count and the percentage of CD4+ T cells of the patients infected with HIV-1 are negatively correlated to the viral load [26,27] and analysis of the correlation of log viral load with CD4+ T cell counts and the percent of CD4+ T cells in a group of drug naïve Kenyan women also showed a linear correlation (Multiple R:0.921, R^2^: 0.848), the similar CD4+ counts for both groups of women in the absence of anti-retroviral treatment (>500) indicate that they were healthy and with comparable viral load at the time of this study. Furthermore, CD4+ and CD8+ T cell counts stratified by different HLA class II phenotypes (data not shown) associated with different outcomes showed no significant difference during the time of this study. In the absence of antiretroviral treatment and with CD4+ counts greater than 500, indicate a relatively health status of these women at the time of this study and the likelihood of other confounding factors is small.

Mother-child transmission has been reduced to less than 1 percent in developed countries with prenatal HIV counselling and testing, antiretroviral prophylaxis, elective caesarean delivery and avoidance of breastfeeding. However, in developing countries where the majority of HIV-infected women live, ART is complex, expensive and is still not available to many. Also, over time, there will be reduced effectiveness due to the development of ARV resistance. Identifying and understanding the genetic factors associated with protection and increased risk will help to develop alternative prevention and treatment methods.

## References

[1] UNAIDS website. Available: http://www.unaids.org. Accessed 2014 Aug 18.

[2] Luzuriaga K, Sullivan JL. Pediatric HIV-1 infection: advances and remaining challenges. AIDS Rev. 2002;4:21–6. 11998780

[3] Scarlatti G. Mother-to-child transmission of HIV-1: advances and controversies of the twentieth centuries. AIDS Rev. 2004;6:67–78. 15332429

[4] Fawzi W, Msamanga G, Renjifo B, Spiegelman D, Urassa E, Hashemi L, et al Predictors of intrauterine and intrapartum transmission of HIV-1 among Tanzanian women. Aids. 2001;15:1157–65. 11416718 10.1097/00002030-200106150-00011

[5] MacDonald KS, Embree JE, Nagelkerke NJ, Castillo J, Ramhadin S, Njenga S, et al The HLA A2/6802 supertype is associated with reduced risk of perinatal human immunodeficiency virus type 1 transmission. J Infect Dis. 2001;183:503–6. 11133384 10.1086/318092

[6] Polycarpou A, Ntais C, Korber BT, Elrich HA, Winchester R, Krogstad P, et al Association between maternal and infant class I and II HLA alleles and of their concordance with the risk of perinatal HIV type 1 transmission. AIDS Res Hum Retroviruses. 2002;18:741–6. 12167265 10.1089/08892220260139477

[7] Aikhionbare FO, Kumaresan K, Shamsa F, Bond VC. HLA-G DNA sequence variants and risk of perinatal HIV-1 transmission. AIDS Res Ther. 2006;3:28 17059603 10.1186/1742-6405-3-28 PMC1634865

[8] Aikhionbare FO, Hodge T, Kuhn L, Bulterys M, Abrams EJ, Bond VC. Mother-to-child discordance in HLA-G exon 2 is associated with a reduced risk of perinatal HIV-1 transmission. Aids. 2001;15:2196–8. 11684943 10.1097/00002030-200111090-00019

[9] Mackelprang RD, John-Stewart G, Carrington M, Richardson B, Rowland-Jones S, Gao X, et al Maternal HLA homozygosity and mother-child HLA concordance increase the risk of vertical transmission of HIV-1. J Infect Dis. 2008;197:1156–61. 18462163 10.1086/529528 PMC2689391

[10] MacDonald KS, Embree J, Njenga S, Nagelkerke NJ, Ngatia I, Mohammed Z, et al Mother-child class I HLA concordance increases perinatal human immunodeficiency virus type 1 transmission. J Infect Dis. 1998;177:551–6. 9498431 10.1086/514243

[11] Gould SJ, Hildreth JE, Booth AM. The evolution of alloimmunity and the genesis of adaptive immunity. Q Rev Biol. 2004;79:359–82. 15669770 10.1086/426088

[12] MacDonald KS, Fowke KR, Kimani J, Dunand VA, Nagelkerke NJ, Ball TB, et al Influence of HLA supertypes on susceptibility and resistance to human immunodeficiency virus type 1 infection. J Infect Dis. 2000;181:1581–9. 10823757 10.1086/315472

[13] Robinson J, Malik A, Parham P, Bodmer JG, Marsh SG. IMGT/HLA database—a sequence database for the human major histocompatibility complex. Tissue Antigens. 2000;55:280–7. 10777106 10.1034/j.1399-0039.2000.550314.x

[14] Robinson J, Waller MJ, Parham P, Bodmer JG, Marsh SG. IMGT/HLA Database—a sequence database for the human major histocompatibility complex. Nucleic Acids Res. 2001;29:210–3. 11125094 10.1093/nar/29.1.210 PMC29780

[15] Robinson J, Waller MJ, Parham P, et al IMGT/HLA and IMGT/MHC: sequence databases for the study of the major histocompatibility complex. Nucleic Acids Res. 2003;31:311–4. 12520010 10.1093/nar/gkg070 PMC165517

[16] Flomenberg N, Baxter-Lowe LA, Confer D, Fernandez-Vina M, Filipovich A, Horowitz M, et al Impact of HLA class I and class II high-resolution matching on outcomes of unrelated donor bone marrow transplantation: HLA-C mismatching is associated with a strong adverse effect on transplantation outcome. Blood. 2004;104:1923–30. 15191952 10.1182/blood-2004-03-0803

[17] Petersdorf EW, Kollman C, Hurley CK, Dupont B, Nademanee A, Begovich AB, et al Effect of HLA class II gene disparity on clinical outcome in unrelated donor hematopoietic cell transplantation for chronic myeloid leukemia: the US National Marrow Donor Program Experience. Blood. 2001;98:2922–9. 11698272 10.1182/blood.v98.10.2922

[18] Petersdorf EW, Gooley TA, Anasetti C, Martin PJ, Smith AG, Mickelson EM, et al Optimizing outcome after unrelated marrow transplantation by comprehensive matching of HLA class I and II alleles in the donor and recipient. Blood. 1998;92:3515–20. 9808542

[19] Santamaria P, Reinsmoen NL, Lindstrom AL, Boyce-Jacino MT, Barbosa JJ, Faras AJ, et al Frequent HLA class I and DP sequence mismatches in serologically (HLA-A, HLA-B, HLA-DR) and molecularly (HLA-DRB1, HLA-DQA1, HLA-DQB1) HLA-identical unrelated bone marrow transplant pairs. Blood. 1994;83:3834 8204903

[20] Hardie RA, Luo M, Bruneau B, Knight E, Nagelkerke NJ, Kimani J, et al Human leukocyte antigen-DQ alleles and haplotypes and their associations with resistance and susceptibility to HIV-1 infection. Aids. 2008;22:807–16. 18427198 10.1097/QAD.0b013e3282f51b71

[21] Lacap PA, Huntington JD, Luo M, Nagelkerke NJ, Bielawny T, Kimani J, et al Associations of human leukocyte antigen DRB with resistance or susceptibility to HIV-1 infection in the Pumwani Sex Worker Cohort. Aids. 2008;22:1029–38. 18520346 10.1097/QAD.0b013e3282ffb3db

[22] Hardie RA, Knight E, Bruneau B, Semeniuk C, Gill K, Nagelkerke N, et al A common human leucocyte antigen-DP genotype is associated with resistance to HIV-1 infection in Kenyan sex workers. Aids. 2008;22:2038–42. 18784467 10.1097/QAD.0b013e328311d1a0 PMC2683274

[23] Datta P, Embree JE, Kreiss JK, Ndinya-Achola JO, Braddick M, Temmerman M, et al Mother-to-child transmission of human immunodeficiency virus type 1: report from the Nairobi Study. J Infect Dis. 1994;170:1134–40. 7963705 10.1093/infdis/170.5.1134

[24] Luo M, Blanchard J, Brunham K, Pan Y, Shen CX, Lu H, et al Two-step high resolution sequence-based HLA-DRB typing of exon 2 DNA with taxonomy-based sequence analysis allele assignment. Hum Immunol. 2001;62:1294–310. 11704293 10.1016/s0198-8859(01)00339-1

[25] Luo M, Blanchard J, Pan Y, Brunham K, Brunham RC. High-resolution sequence typing of HLA-DQA1 and-DQB1 exon 2 DNA with taxonomy-based sequence analysis (TBSA) allele assignment. Tissue Antigens. 1999;54:69–82. 10458325 10.1034/j.1399-0039.1999.540108.x

[26] Ho BC, Laudette-Aboulhab J, Se-Thoe SY, Chan SP, Ling AE, Leo YS. Correlation of baseline quantitative plasma human immunodeficiency (HIV) type 1 RNA viral load with clinical status and CD4+ T-cell counts in treatment-naive HIV-positive patients in Singapore. Ann Acad Med Singapore. 2000;29:708–13. 11269974

[27] Kannangai R, Ramalingam S, Jesudason MV, Vijayakumar TS, Abraham OC, Zachariah, et al Correlation of CD4(+) T-Cell counts estimated by an immunocapture technique (Capcellia) with viral loads in human immunodeficiency virus-seropositive individuals. Clin Diagn Lab Immunol. 2001;8:1286–8. 11687479 10.1128/CDLI.8.6.1286-1288.2001 PMC96265

[28] Robinson J, Marsh SG. The IMGT/HLA sequence database. Rev Immunogenet. 2000;2:518–31. 12361093

[29] Robinson J, Waller MJ, Fail SC, Marsh SG. The IMGT/HLA and IPD databases. Hum Mutat. 2006;27:1192–9. 16944494 10.1002/humu.20406

[30] Blanck G. HLA class II expression in human tumor lines. Microbes Infect. 1999;1:913–8. 10614009 10.1016/s1286-4579(99)00226-9

[31] Alcaide-Loridan C, Lennon AM, Bono MR, Barbouche R, Dellagi K, Fellous M. Differential expression of MHC class II isotype chains. Microbes Infect. 1999;1:929–34. 10614011 10.1016/s1286-4579(99)00224-5

[32] Tang J, Penman-Aguilar A, Lobashevsky E, Allen S, Kaslow RA. HLA-DRB1 and-DQB1 alleles and haplotypes in Zambian couples and their associations with heterosexual transmission of HIV type 1. J Infect Dis. 2004;189:1696–704. 15116308 10.1086/383280

[33] Selvaraj P, Swaminathan S, Alagarasu K, Raghavan S, Narendran G, Narayanan P. Association of human leukocyte antigen-A11 with resistance and B40 and DR2 with susceptibility to HIV-1 infection in south India. J Acquir Immune Defic Syndr. 2006;43:497–9. 17099315 10.1097/01.qai.0000233312.36226.76

[34] Prat E, Tomaru U, Sabater L, Park DM, Granger R, Kruse N, et al HLA-DRB5*0101 and-DRB1*1501 expression in the multiple sclerosis-associated HLA-DR15 haplotype. J Neuroimmunol. 2005;167:108–19. 16111772 10.1016/j.jneuroim.2005.04.027

[35] Debaz H, Olivo A, Vazquez Garcia MN, de la Rosa G, Hernandez A, Lino L, et al Relevant residues of DRbeta1 third hypervariable region contributing to the expression and to severity of rheumatoid arthritis (RA) in Mexicans. Hum Immunol. 1998;59:287–94. 9619767 10.1016/s0198-8859(98)00017-2

[36] Hader SL, Hodge TW, Buchacz KA, Bray RA, Padian NS, Rausa A, et al Discordance at human leukocyte antigen-DRB3 and protection from human immunodeficiency virus type 1 transmission. J Infect Dis. 2002;185:1729–35. 12085318 10.1086/340648

[37] Sampathkumar R, Peters HO, Mendoza L, Bielawny T, Ngugi E, Kimani J, et al Influence of HLA class I haplotypes on HIV-1 seroconversion and disease progression in Pumwani sex worker cohort. PloS One 2014 7 3; 9(7):e101475 doi: 10.1371/journal.pone.0101475 24992306 10.1371/journal.pone.0101475 PMC4081595

